# The association between adaptability and the symptoms of depression and anxiety in early adolescents: a network analysis in a longitudinal design

**DOI:** 10.1186/s12888-025-06559-z

**Published:** 2025-02-12

**Authors:** Gangyuan Lu, Linfei Zhu, Rongqian Huang, Pengcheng Lai, Chao Wang

**Affiliations:** 1 School of Psychology, Shenzhen University, Shenzhen, 518060 Guangdong China; 2 Nanshan Taoyuan Primary School, Shenzhen, 518055 Guangdong China

**Keywords:** Network analysis, Adaptability, Depression, Anxiety, Adolescent

## Abstract

**Background:**

The co-occurrence of depression and anxiety is relatively more common among adolescents. Emerging evidence suggests that adaptability might affect this phenomenon. Network analysis can provide insight into the dynamics between symptoms of mental disorders. Therefore, we used network analysis 1) to explore symptom networks and 2) to investigate the association between adaptability and symptoms based on a longitudinal design.

**Methods:**

833 Chinese adolescents (449 males and 384 female) were recruited, with adaptability, depressive and anxiety symptoms measured at T1, 426 of them were followed up one year later at T2. Symptom networks were constructed for all participants and for the two groups based on their adaptability scores at T1. Furthermore, mediation analysis was performed to examine the relationship between adaptability and bridge symptoms at both timepoints.

**Results:**

Irritable and Guilty showed the highest expected value at T1 (*p* < 0.05). The high adaptability group’s symptom network (HGN) was less connected than the low adaptability group’s symptom network (LGN) (*p* < 0.001). Furthermore, Irritable (T1) and adaptability (T2) were sequential mediators (*p* < 0.001) between adaptability (T1) and Irritable (T2).

**Conclusions:**

These findings suggest that adaptability might affect the network dynamics, underscoring its importance to the occurrence of depression and anxiety among adolescents. Irritable and Guilty being the bridge symptoms may indicate the interventions to target in adolescents with comorbid depression and anxiety.

**Supplementary Information:**

The online version contains supplementary material available at 10.1186/s12888-025-06559-z.

## Introduction

In the past decade, the prevalence of depression and anxiety in adolescents has sharply increased [1], which causing a range of serious health and social problems including substance abuse (e.g., alcoholism, drug use) and self-harm among adolescents [2–5]. The comorbidity rate of depression and anxiety is relatively higher in children and adolescents, over 95% of children with depression have comorbid conditions, with the highest rate for anxiety [6]. Compared with a single diagnosis of depression or anxiety, individuals are often diagnosed with more than two psychiatric illnesses. The comorbidity is associated with more severe cognitive and social functioning impairments, a greater decline in response to treatment and a higher risk of suicide [7, 8]. Therefore, the co-occurrence of depressive and anxiety symptoms is prevalent among adolescents, and identifying its risk factors is important for both individuals and society.

One potential reason for the relatively high incidence of depressive and anxiety symptoms among adolescents might be poor adaptation to various environmental changes. A study on American adolescents revealed that the first onset of depression occurs around the age of 15, which is when most adolescents have just entered high school [9]. Other studies from China found that freshmen in high school have higher scores of depression and anxiety compared to those in higher grades [10, 11]. Adaptability refers to the ability to adjust one’s cognition, emotions, and behavior in response to a new environment [12]. It was negatively associated with depression and anxiety among college students [13, 14]. In a broader sense, adaptability also manifests as academic resilience. A supportive environment can foster adaptability [15], enabling individuals to successfully cope with adverse circumstances [16]. Some studies have found that intervention methods targeting the enhancement of adaptability can improve an individual’s mental health, such as reducing anxiety and internet addiction [17, 18]. Therefore, these findings imply that adaptability may be a crucial factor affecting the symptoms of depression and anxiety among school-aged adolescents.

According to the network theory of psychopathology, mental disorders may be a series of symptoms that occur simultaneously, and these symptoms can interact with and reinforce each other [19, 20]. For instance, anxiety is a common trigger for depression [21], and conversely, depression can also increase the risk of anxiety [1]. Network analysis offers a direct insight into the associations between symptoms of two different mental disorders by treating them as nodes and their connections as edges. It assumes that comorbidity may arise when some key symptoms, or “bridge symptoms,” in one mental disorder trigger symptoms in another [19]. Most network analyses have been employed to understand the networks of depressive and anxiety symptoms in adults [22–24], but only a few studies have focused on adolescents [25, 26]. However, adolescents and adults exhibit different clinical features related to depression and anxiety [3, 4], so there is insufficient basis to presume that findings of network analysis in adults can be directly applied to adolescents experiencing these symptoms. Furthermore, a few studies have launched exploratory research on the network relationships between symptoms of depression and anxiety in adults and adolescents, yet the results have been quite inconsistent [27–29]. Longitudinal sampling will allow us to examine how the relationship between adaptability, depression and anxiety symptoms operates over time in adults [30]. Additionally, these studies have not taken into account the possible factors influencing the networks via bridge symptoms.

Hence, the current study aimed to investigate the symptom network and explore the association between adaptability and the symptoms of depression and anxiety among adolescents through a longitudinal design. We hypothesized that adaptability was negatively related to depression and anxiety, and its potential influence on the network. Network analyses were conducted to identify crucial symptoms that contribute to the co-occurrence of depression and anxiety. Network comparisons would be conducted based on cross-sectional and longitudinal symptom networks. Furthermore, the mediation analysis would be performed to explore the relationships between adaptability and changes in key symptoms.

## Methods

### Participants

461 students in Grade 7 and 372 students in Grade 10 were recruited from a school in China, 833 participants in total (384 females and 449 males; age: mean ± SD = 13.67 ± 1.52). Only 426 students in Grade 7 were followed up one year later at T2, while others withdrew from the study due to academic arrangements and relatively low participation willingness at T2, resulting in unsuccessful follow-up.

### Measures

The severity of depression and anxiety was assessed using the Patient Health Questionnaire-8 (PHQ-8) [31] and Generalized Anxiety Disorder-7 (GAD-7) [32]. PHQ-8 includes 8 items: Anhedonia, Mood, Sleep, Energy, Appetite, Guilty, Concentration, and Motor, while GAD-7 includes 7 items: Nervous, Control worry, Too much worry, Relax, Restless, Irritable, and Afraid. These items are scored from 0 (not at all) to 3 (nearly every day), with higher scores indicating more severe symptoms. Adaptability was measured using the Adaptation Scale for Adolescents (ASA) (D. [33]). ASA defines adaptation as the psychological and behavioral adjustment to physiological, social, life, interpersonal, academic, and emotional changes, with its 22 items measuring adaptability across these six dimensions. Each item is scored from 1 (not at all) to 4 (completely matched). Adaptability scores were calculated by averaging the scores of all items, with higher scores indicating better adaptability. The reliability of the above three scales in this study were measured using Cronbach’s alpha coefficient, which was 0.84, 0.88 and 0.92, respectively.

### Data analysis

All analyses were performed in R software (v 4.3.2) [34]. The symptom networks at T1 and T2 were constructed using the bootnet package [35] and visualized using the qgraph package [36]. The Fruchterman-Reingold algorithm places important nodes at the center of the graph and trivial ones at the periphery.

Gaussian Graphical Model (GGM) was employed to estimate the symptom networks, wherein nodes represent symptoms and edges denote the associations between them. Due to the skewed distributions of item scores, which does not conform to the assumption of GGM [35], a nonparanormal transformation was conducted before GGM network analysis to address this issue, during which the nonparametric correlations were calculated [22],H. [37]. The weight of an edge reflects the magnitude of the nonparametric correlation coefficient, with a higher weight indicating a greater coefficient. To quantify the importance of nodes, we computed four common centrality indices: strength, expected influence (EI), closeness, and betweenness. However, since the estimated networks contained negative edges, we only focused on EI, which represents a node’s connectivity with others. Regarding model selection, we employed the regularization technique, which removes spurious edges using the graphical Least Absolute Shrinkage and Selection Operator (LASSO), to estimate a more concise and interpretable network. The simplicity of the estimated network is determined by the Extended Bayesian Information Criterion (EBIC) [38]. To better display differences between the compared networks, the `averageLayout` function was used to place identical nodes at the same locations on the graph.

The mgm package [39] was used to compute predictability for each symptom. Predictability indicates the extent to which the variance of a symptom in the network can be explained by the variance of its connected symptoms, reflecting the extent to which the symptom can be indirectly influenced by intervening with other symptoms. The mean predictability of the network represents how sensitive the network is to external factors, with a lower value indicating greater influence from external factors.

To identify bridge symptoms, bridge centrality was computed using the network tools package [19]. Here we focused on 1-step bridge expected influence (bridge EI), which reflects the extent to which a symptom acts as a bridge linking two communities.

For testing the reliability of the networks, the non-parametric bootstrapping with 2500 samples was employed via the `bootnet` function for examining the differences and stability of centrality indices and edge weights [35]. The stability of centrality indices was evaluated using a case-dropping procedure, which involves re-estimating the network with a reduced sample to observe whether the node ranking of centrality indices (e.g., EI) changes. The Correlation Stability (CS) coefficient quantifies this stability, indicating the maximum proportion of cases from the original sample that can be dropped while maintaining a correlation of 0.70 or above for centrality indices with 95% probability. A CS coefficient below 0.25 is unacceptable as it renders interpretations involving centrality indices unreliable.

Participants were divided into high and low adaptability groups based on the mean of their adaptability scores. Then, the global and local differences between the symptom networks of the two groups were compared using the Network Comparison Test package [40]. Moreover, the differences between the symptom networks at T1 and T2 were also compared. The global differences involve the structure and global strength between networks, while the local differences concern the identical edges.

Mediation analysis in PROCESS for SPSS (version 4.3) was performed to explore the relationships between adaptability and Irritable based on the data collected at both timepoints [41]. Model 6 was selected, with 1000 bootstrap samples and 95% confidence intervals (CIs). Gender and age at T1 were entered as covariates. 8 participants were excluded due to their adaptability scores at T1 or T2, or the difference between them, exceeded 3 standard deviations from the mean, leading to 418 participants’ data for the mediation analysis.

## Results

### Adaptability, depression, and anxiety scores at T1 and T2

The scores for adaptability, depression, and anxiety were all approximately normally distributed (Table S1) (Kline, 1998). At T1, the high adaptability group had higher adaptability scores and lower depression and anxiety scores compared to the low adaptability group (Table 1). Correlation analysis showed that adaptability scores were negatively associated with both depression and anxiety scores (Depression:* r* = −0.629, *p* = 6.97 × 10^–93^; Anxiety: *r* = −0.583, *p* = 4.60 × 10^–77^, while depression and anxiety scores were positively correlated (*r* = 0.778, *p* = 1.39 × 10^–169^). Only 426 participants (female/male = 206/220) were involved in the questionnaire survey at T2 one year later. Paired sample t-test revealed that adaptability scores at T2 were lower than at T1, while depression and anxiety scores were higher (Table 1).

**Table 1:** Adaptability, depression, and anxiety scores

	High (*N* = 402)	Low (*N* = 431)	Whole (*N* = 833)	*t*	T1 (*N* = 426)	T2 (*N* = 426)	*t*
Adaptability	3.49 ± 2.92	2.68 ± 2.95	3.07 ± 0.50	39.46^***^	3.11 ± 0.52	3.03 ± 0.60	−3.04^***^
Depression	2.96 ± 2.79	7.52 ± 4.88	5.32 ± 4.61	−16.67^***^	4.47 ± 4.12	5.76 ± 5.18	5.95^***^
Anxiety	2.03 ± 2.62	6.04 ± 4.71	4.11 ± 4.34	−15.34^***^	3.50 ± 4.05	4.77 ± 5.10	5.96^***^

Mean ± Standard deviation, High = The high adaptability group at T1, Low = The low adaptability group at T1, Whole = All participants at T1, T1 = The first timepoint of measurement, T2 = The second timepoint of measurement. ^***^p < 0.001

### The symptom networks at T1

**Fig. 1:**
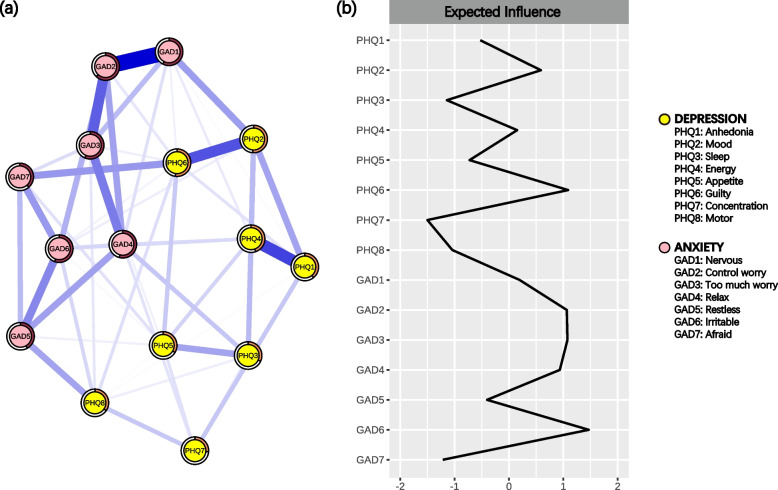
The symptom network for all participants at T1 (*N* = 833). **a** The symptom network structure. Yellow nodes represent the symptoms of depression; pink nodes represent the symptoms of anxiety disorder. A wider edge signifies a greater weight between nodes. The ring-shaped pie charts represent the predictability. **b** The expected influence for each node. This index reflects the importance of a node in the network. The values are Z‐standardized

The whole symptom network (WCN) of depression and anxiety for all participants at T1 had 81 edges out of 105 possible ones, resulting in a density of 0.77 (Table S2 and Fig. S1). The three strongest symptoms were Irritable (EI = 1.11), Guilty (EI = 1.05), and Too much worry (EI = 1.05) (Fig. 1 and S2). The three strongest edges were Nervous-Control worry (weight = 0.33), Anhedonia-Energy (weight = 0.26), and Mood-Guilty (weight = 0.24) (Fig. S3). The mean predictability of WCN was 45.34%. The three symptoms with the highest predictability were Control worry (predictability = 62.07%), Nervous (predictability = 57.59%), and Too much worry (predictability = 56.44%), which all belonged to the anxiety community (Table S3).

Then, HGN and LGN were constructed separately (Fig. S4). The density of these networks was 0.68 and 0.75 (Table S4-5 and Fig. S1), with the mean predictability being 32.94% and 42.15%, respectively (Table S3). The CS coefficient of EI was above 0.50 for both WCN and LGN (0.75 and 0.59, respectively), while for HGN it was 0.44, indicating that the estimates for EI were reliable (Fig. S5).

### Identifying bridge symptoms linking the two communities at T1

**Fig. 2:**
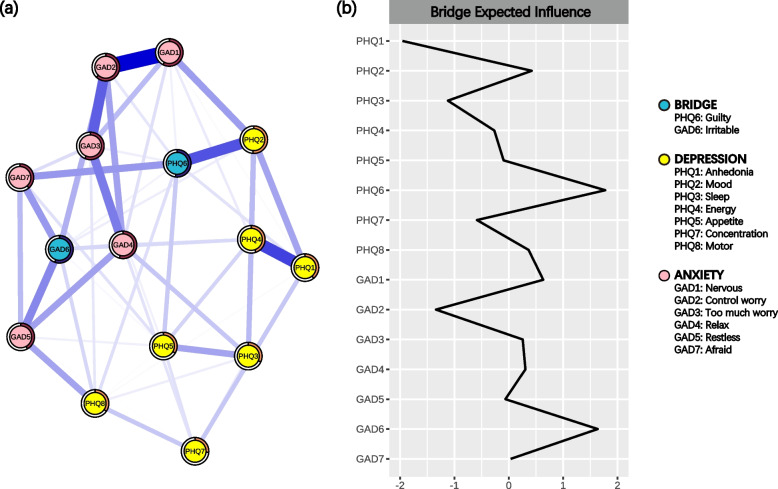
The symptom network for all participants at T1 showing the bridge symptoms (*N* = 833). **a** The symptom network structure. Yellow nodes represent the symptoms of depression; pink nodes represent the symptoms of anxiety disorder; blue nodes represent the bridge symptoms. Irritable and Guilty are the bridge symptoms. The ring-shaped pie charts represent the predictability. **b** The bridge expected influence for each node. This index reflects the extent to which a node acts as a bridge symptom. The values are Z‐standardized

The bridge symptoms were Guilty and Irritable in the three symptom networks mentioned above (Fig. 2 and S6-7). The stability test for bridge EI revealed a CS coefficient of 0.52 for WCN, while the CS coefficient for HGN and LGN was 0.44 and 0.36, respectively (Fig. S5). These results suggest that the estimates for bridge EI were reliable.

### The effect of adaptability on networks at T1

**Fig. 3:**
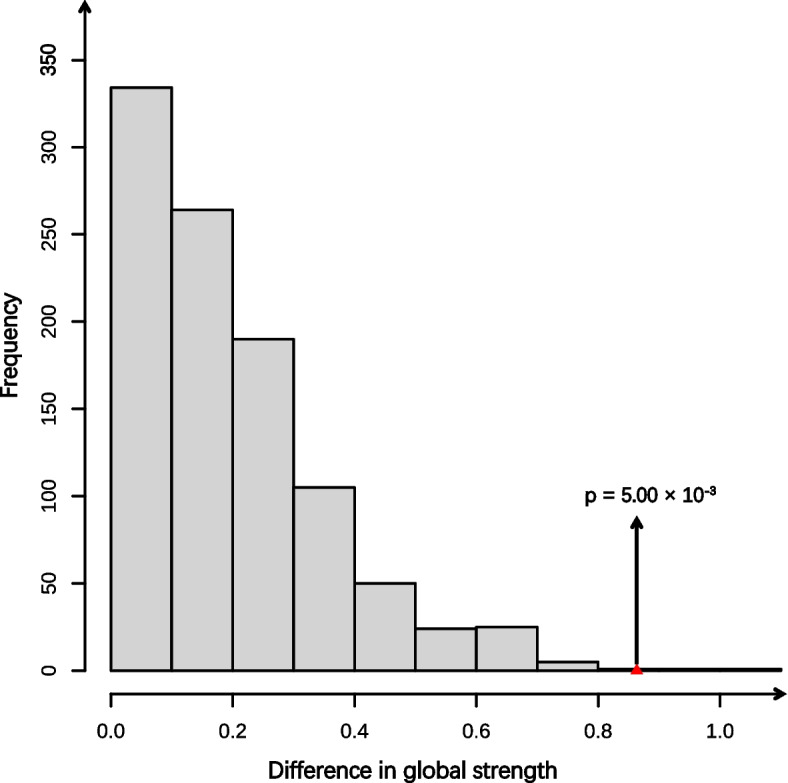
The difference test result for global strength between HGN and LGN. The red triangle indicates the difference in global strength between the two groups. The black arrow points toward the corresponding *p*-value. HGN = The high adaptability group’s symptom network, LGN = The low adaptability group’s symptom network

After network comparison, we found that the global strength of HGN was lower than that of LGN (HGN: 5.90, LGN: 6.76, *p* = 5.00 × 10^–3^, Fig. 3). No significant difference was observed in network structure, and none of the edges showed significant differences between two networks after the false discovery rate (FDR) correction (Table S6). However, in HGN, Sleep exhibited a lower EI compared to LGN, even after the correction (HGN: 0.45, LGN: 0.77, *p* = 0.01, Table S7).

### The impact of adaptability on network properties based on longitudinal data

Considering the relative change in sample size, we constructed the symptom networks and identified the bridge symptoms at both timepoints and conducted a network comparison between them (Table S8-12 and Fig. S8-14). The bridge symptoms at T1 were Guilty and Irritable, while at T2 they were Motor and Nervous (Fig. S13). Only the bridge EI of Irritable at T2 was relatively lower (T1: 0.58, T2: 0.33, *p*_*uncorrected*_ = 0.03, Table S12). No significant differences were observed in network structure and global strength between the two timepoints. Additionally, none of the edges exhibited significant differences after FDR correction.

**Fig. 4:**
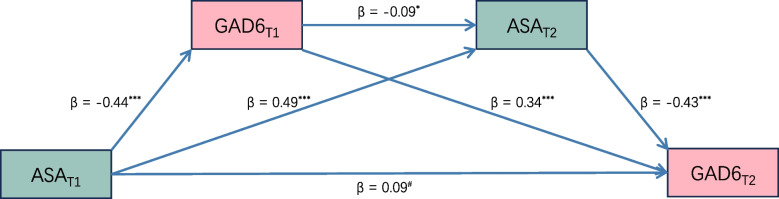
The serial mediation model. The model depicts the relationships between adaptability (ASA) and Irritable (GAD6) at both timepoints of measurement (T1 and T2). *β* = The standardized regression coefficient, ^#^*p* > 0.05, ^*^p < 0.05, ^***^p < 0.001

To investigate the potential correlation between adaptability and the decline EI of Irritable, a serial mediation model was used. We found that the Irritable (T1) and the adaptability (T2) were sequential mediators between the adaptability (T1, predictor) and Irritable (T2, outcome) (Fig. 4). The detailed results were: 1) Irritable (T1) mediated the association between adaptability (T1) and Irritable (T2) (indirect effect = −0.15, 95% CI [−0.20, −0.10]); 2) adaptability (T2) mediated the association between adaptability (T1) and Irritable (T2) (indirect effect = −0.21, 95% CI [−0.28, −0.15]); 3) Irritable (T1) and adaptability (T2) sequentially mediated the association between adaptability (T1) and Irritable (T2) (indirect effect = −0.02, 95% CI [−0.04, −1.30 × 10^–3^]).

## Discussion

Our study offered a novel insight into the relationship between adaptability and the symptoms of depression and anxiety. Irritable and Guilty were the most central symptoms and linked the two communities at T1. We found that the global strengths between HGN and LGN were significantly different and the bridge EI of Irritable decreased at T2 compared to T1. Importantly, we identified that adaptability may exert influence on the network of depressive and anxiety symptoms through Irritable in the serial mediation model.

Consistent with our previous speculation, adaptability was negatively associated with both depression and anxiety among school-aged adolescents [13, 14]. The high adaptability group had lower depression and anxiety scores than the low adaptability group. According the transactional model of stress and coping [42], successful coping requires the ability to adjust and change coping strategies to more effectively get positive results. Adolescents with high adaptability are more likely to have positive coping responses, such as solving problems rationally and avoiding self-deprecation, and the result of successful coping means better adaptation, higher resilience [43]. This, in turn, reduces the chance of developing depressive and anxiety symptoms [13, 14, 44].

Interestingly, Irritable was the bridge symptom at T1, while the bridge EI of Irritable decreased at T2. Previous studies have identified Irritable as a central symptom in the symptom network [22–24]. Diagnostic and Statistical Manual of Mental Disorders (DSM-5TM, 5th Ed., 2013) has also recognized Irritable as a diagnostic criterion for both depression and anxiety. An abnormal level of irritability in both mental illnesses may be attributed to heredity. For instance, the level of irritability, along with symptoms of both depression and anxiety, can be influenced by the same genes [45]. However, the bridge EI of Irritable decreased at T2 compared to T1. We speculated that the diminished connections between Irritable and other symptoms were the underlying reason. Indeed, a decrease was observed in the associations between Irritable and Sleep, Energy, and Appetite at T2. Similar associations have been reported in other studies [20, 23, 24]. Furthermore, mediation analysis revealed that an increase in adaptability at T1 reduced the level of irritability, which further enhanced adaptability at T2, consequently lowering the level of irritability at T2. This finding is consistent with a study showing that difficulties in emotion regulation positively predict aggression level among adolescents [17, 46]. The development of dorsolateral prefrontal cortex (DLPFC) is closely associated with an increase in execution function, which enhances emotion regulation [47]. Adolescents with higher adaptability at T1 exhibited effective emotion regulation, possibly due to the earlier development of their DLPFC compared to their peers. This development may boost emotion regulation, resulting in a reduced level of irritability at T2. In summary, these findings suggest that adaptability could influence the network of depressive and anxiety symptoms via Irritable, highlighting its significance to adolescents.

Remarkably, HGN showed a lower global strength compared to LGN. Global strength is obtained by summing the absolute values of all edge weights, reflecting the connectivity level of a network. A higher value indicates a more closely connected network [40]. HGN was relatively sparse, possibly because adaptability diminished the relationships between symptoms. For instance, emotion regulation moderates the associations between insomnia severity and anxiety symptoms [48]. Furthermore, Sleep showed a greater EI in LGN compared to HGN. Negative coping styles are correlated with lower sleep quality [49]. Some studies suggest that insomnia is a risk factor for both depression and anxiety [50, 51]. Adolescents with low adaptability may struggle to address stressful problems or indulge in negative emotions, making them more likely to experience insomnia, thereby increasing the co-occurrence of depression and anxiety.

Additionally, Guilty as a bridge symptom also aligns with previous findings [26, 52], which could be attributed to the high expectations Chinese students face from elders. Adolescents may tend to be more self-deprecating when they are unable to fulfill these expectations, leading to the emergence of other depressive symptoms and a higher level of anxiety [53–55]. Motor as a bridge symptom is in line with some studies [20, 56]. Patients diagnosed with both mental illnesses are more physically active than those only diagnosed with depression or anxiety [57]. Furthermore, Nervous as a bridge symptom may be attributed to the challenging curriculum in a higher grade, causing increased concerns about academic performance. In conclusion, these findings underscore the importance of both physical and mental health in the context of adolescent well-being.

Within the symptom networks at T1, the strongest edge was Nervous-Control worry, aligning with several findings [23, 24, 52]. The former item measures participants’ emotion, while the latter one indirectly reflects their ability of emotion regulation. When adolescents struggle to control their anxiety, they tend to exhibit nervousness [44]. This interpretation is supported by one study which found a high redundancy between Nervous and Control worry [58]. Within the symptom network at T2, Too much worry remained strong, while the strongest edge was Control worry-Too much worry. An increase in academic pressure may be the reason for this shift. Specifically, as adolescents entering a higher grade, the curriculum becomes more demanding, which may trigger their persistent worries [11]. When such worries intensify, adolescents may struggle to manage their emotional turmoil, leading to excessive anxiety [29].

In line with another study [24], Mood-Guilty and Anhedonia-Energy are two strong edges. Mood and Guilty belong to the same sub-community [20] and guilt is associated with mood disturbance [59], explaining their close connection in the network. Regarding Anhedonia-Energy, one study revealed that about 42% of adolescents with chronic fatigue syndrome report experiencing anhedonia [60]. Similarly, fatigue still accounts for 20% of the variance in anhedonia even after controlling for depression in the schizophrenic population [61]. It is possible that the connection between these two symptoms may arise from some common underlying mechanisms. For instance, reduced activation in the striatum is associated with an increase in both anhedonia and fatigue [62]. Taken together, these findings suggest that it is worthwhile for researchers to explore the mechanisms under these closely connected symptoms, facilitating the clinical treatment.

Control worry and Too much worry exhibited the highest predictability at T1 and T2, consistent with previous findings [24, 56]. We observed strong connections between Relax and Control worry and Too much worry, which implies that we could indirectly intervene with these symptoms through Relax. For example, a review showed the efficacy of relaxation training in alleviating anxiety [63]. Thus, these findings suggest that relaxation training such as breathing meditation [64] could be employed in clinical treatments to indirectly alleviate patients’ worry, thereby enhancing the efficacy of treatments.

Our findings have significant implications for clinical treatment and policy in educational institutions. First, Irritable and Guilty are bridge symptoms with the highest expected influence in the whole symptom network, which emphasizes their priority for treatment in the future clinical practice. Second, HGN was less connected than LGN, suggesting that ASA combined with other scales, such as Cyber-Bullying /Victimization Experience Questionnaire [65], can used by staff in educational institutions to identify potential mental disorders and plan interventions to prevent their progression. Finally, the mediating role of Irritable between adaptability and the network highlights the need for mental health education programs that teach students how to manage negative emotions and foster adaptability to cope with academic challenges. Overall, this research underscores the importance for policymakers to invest more in mental health initiatives, which can improve students’ well-being.

Our study had several limitations. First, the sample size was modest. To mitigate this problem, regularization was employed to select an optimal model [30], a technique known for accurately estimating network even with a relatively small sample size. The stability tests further affirmed the robustness of our estimates, minimizing the concern about the sample size. Second, the inclusion of only Chinese adolescents may limit the generalizability of the findings, underscoring the need for recruiting adolescents from diverse regions in the future research. Third, our study did not explore the causal relationships between symptoms. Estimating a directed network requires more measurements compared to constructing an undirected network. Given that we only had the data collected from both timepoints, estimating a directed network is not practical. Therefore, one future direction for our research is to gather larger longitudinal data to construct the directed network, providing further evidence for the conclusions drawn here. Fourth, we were unable to track the Grade 10 students at T2 due to their academic arrangements and low willingness. If there is an opportunity in the future, we will supplement the research on the Grade 10 to improve our understanding of this field. Fifth, previous studies have suggested that pubertal level is a risk factor for both depression and anxiety [66–68]. However, this variable was not measured in our study. Including it may be necessary for future research. Sixth, our study relied on self-reported assessments to collect a large sample, which may not capture some detailed information that could be obtained through face-to-face interviews. Finally, the networks constructed in this study are specific to the scales used. There may be some inconsistency when applying other questionnaires, suggesting a future direction for comparing networks constructed from different scales measuring the same mental disorder.

## Conclusions

To the best of our knowledge, our study is the first to investigate the effect of adaptability on the symptom network. Irritable and Guilty were crucial symptoms within the symptom network. Notably, adaptability could influence the network of depressive and anxiety symptoms via Irritable. These findings suggest that the comorbidity rate of depression and anxiety may decrease by improving adolescents’ adaptability, thereby promoting their mental health.

## Supplementary Information

Supplementary Material 1.

## Data Availability

The data that support the findings of this study are available from the corresponding author upon reasonable request.
