# Supplementary material for: The association between adaptability and the symptoms of depression and anxiety in early adolescents: a network analysis in a longitudinal design
Source: BMC Psychiatry. 2025 Feb 12;25:117. doi: 10.1186/s12888-025-06559-z (PMC11817081; doi:10.1186/s12888-025-06559-z)
Supplement: Supplementary file 1 — Supplementary Material 1. [file 12888_2025_6559_MOESM1_ESM.docx]

Table S1 The skewness and kurtosis of adaptability, depression, and anxiety scores.

|  | Skewness | | | | | Kurtosis | | | | |
| --- | --- | --- | --- | --- | --- | --- | --- | --- | --- | --- |
|  | High | Low | Whole | T1 | T2 | High | Low | Whole | T1 | T2 |
| Adaptability | 0.28 | -1.00 | -0.05 | -0.18 | -0.08 | -1.23 | 1.07 | -0.42 | -0.51 | -0.42 |
| Depression | 1.36 | 0.99 | 1.30 | 1.35 | 1.23 | 3.03 | 1.12 | 2.05 | 2.28 | 1.52 |
| Anxiety | 1.90 | 0.87 | 1.34 | 1.44 | 1.36 | 3.97 | 0.30 | 1.48 | 1.75 | 1.50 |

*Note.* High = The high adaptability group at T1, Low = The low adaptability group at T1, Whole = All participants at T1, T1 = The first timepoint of measurement, T2 = The second timepoint of measurement.

Table S2 The matrix of edge weights of the whole symptom network.

|  | PHQ1 | PHQ2 | PHQ3 | PHQ4 | PHQ5 | PHQ6 | PHQ7 | PHQ8 | GAD1 | GAD2 | GAD3 | GAD4 | GAD5 | GAD6 | GAD7 |
| --- | --- | --- | --- | --- | --- | --- | --- | --- | --- | --- | --- | --- | --- | --- | --- |
| PHQ1 |  |  |  |  |  |  |  |  |  |  |  |  |  |  |  |
| PHQ2 | 0.15 |  |  |  |  |  |  |  |  |  |  |  |  |  |  |
| PHQ3 | 0.05 | 0.13 |  |  |  |  |  |  |  |  |  |  |  |  |  |
| PHQ4 | 0.26 | 0.13 | 0.06 |  |  |  |  |  |  |  |  |  |  |  |  |
| PHQ5 | 0.00 | 0.00 | 0.15 | 0.10 |  |  |  |  |  |  |  |  |  |  |  |
| PHQ6 | 0.08 | 0.24 | 0.00 | 0.05 | 0.11 |  |  |  |  |  |  |  |  |  |  |
| PHQ7 | 0.11 | 0.00 | 0.07 | 0.02 | 0.08 | 0.02 |  |  |  |  |  |  |  |  |  |
| PHQ8 | 0.05 | 0.00 | 0.07 | 0.03 | 0.05 | 0.09 | 0.11 |  |  |  |  |  |  |  |  |
| GAD1 | 0.05 | 0.16 | 0.02 | 0.07 | 0.00 | 0.07 | 0.01 | -0.03 |  |  |  |  |  |  |  |
| GAD2 | 0.00 | 0.05 | 0.01 | 0.00 | 0.00 | 0.08 | 0.02 | 0.00 | 0.33 |  |  |  |  |  |  |
| GAD3 | 0.01 | 0.05 | 0.00 | 0.00 | 0.07 | 0.07 | 0.04 | 0.08 | 0.13 | 0.23 |  |  |  |  |  |
| GAD4 | 0.00 | 0.00 | 0.11 | 0.09 | 0.00 | 0.01 | 0.08 | 0.02 | 0.09 | 0.15 | 0.20 |  |  |  |  |
| GAD5 | 0.01 | 0.00 | 0.00 | 0.00 | 0.07 | 0.00 | 0.05 | 0.15 | 0.00 | 0.04 | 0.04 | 0.16 |  |  |  |
| GAD6 | 0.02 | 0.07 | 0.01 | 0.09 | 0.06 | 0.06 | 0.03 | 0.10 | 0.02 | 0.14 | 0.06 | 0.10 | 0.18 |  |  |
| GAD7 | 0.00 | 0.00 | 0.03 | 0.00 | 0.08 | 0.17 | 0.00 | 0.01 | 0.00 | 0.00 | 0.09 | 0.03 | 0.13 | 0.17 |  |

*Note.* PHQ1 = Anhedonia, PHQ2 = Mood, PHQ3 = Sleep, PHQ4 = Energy, PHQ5 = Appetite, PHQ6 = Guilty, PHQ7 = Concentration, PHQ8 = Motor, GAD1 = Nervous, GAD2 = Control worry, GAD3 = Too much worry, GAD4 = Relax, GAD5 = Restless, GAD6 = Irritable, GAD7 = Afraid.

Table S3 The predictability for each node in the three networks at T1.

|  | HGN  (N = 402) | LGN  (N = 431) | WGN  (N = 833) |
| --- | --- | --- | --- |
| PHQ1 | 0.2736 | 0.3304 | 0.3911 |
| PHQ2 | 0.3782 | 0.4624 | 0.5107 |
| PHQ3 | 0.1349 | 0.3410 | 0.3196 |
| PHQ4 | 0.3110 | 0.3979 | 0.4474 |
| PHQ5 | 0.2306 | 0.3234 | 0.3355 |
| PHQ6 | 0.4019 | 0.4673 | 0.5206 |
| PHQ7 | 0.1288 | 0.2381 | 0.2640 |
| PHQ8 | 0.2464 | 0.3295 | 0.3468 |
| GAD1 | 0.4810 | 0.5306 | 0.5759 |
| GAD2 | 0.5485 | 0.5702 | 0.6207 |
| GAD3 | 0.4111 | 0.5353 | 0.5644 |
| GAD4 | 0.4282 | 0.4978 | 0.5425 |
| GAD5 | 0.2991 | 0.4048 | 0.4198 |
| GAD6 | 0.4463 | 0.4962 | 0.5551 |
| GAD7 | 0.2208 | 0.3974 | 0.3869 |

*Note.* HGN = The high adaptability group’s symptom network, LGN = The low adaptability group’s symptom network, WGN = The whole symptom network, PHQ1 = Anhedonia, PHQ2 = Mood, PHQ3 = Sleep, PHQ4 = Energy, PHQ5 = Appetite, PHQ6 = Guilty, PHQ7 = Concentration, PHQ8 = Motor, GAD1 = Nervous, GAD2 = Control worry, GAD3 = Too much worry, GAD4 = Relax, GAD5 = Restless, GAD6 = Irritable, GAD7 = Afraid.

Table S4 The matrix of edge weights of the high adaptability group’s symptom network.

|  | PHQ1 | PHQ2 | PHQ3 | PHQ4 | PHQ5 | PHQ6 | PHQ7 | PHQ8 | GAD1 | GAD2 | GAD3 | GAD4 | GAD5 | GAD6 | GAD7 |
| --- | --- | --- | --- | --- | --- | --- | --- | --- | --- | --- | --- | --- | --- | --- | --- |
| PHQ1 |  |  |  |  |  |  |  |  |  |  |  |  |  |  |  |
| PHQ2 | 0.16 |  |  |  |  |  |  |  |  |  |  |  |  |  |  |
| PHQ3 | 0.09 | 0.09 |  |  |  |  |  |  |  |  |  |  |  |  |  |
| PHQ4 | 0.24 | 0.09 | 0.05 |  |  |  |  |  |  |  |  |  |  |  |  |
| PHQ5 | 0.00 | 0.04 | 0.07 | 0.10 |  |  |  |  |  |  |  |  |  |  |  |
| PHQ6 | 0.09 | 0.19 | 0.00 | 0.00 | 0.04 |  |  |  |  |  |  |  |  |  |  |
| PHQ7 | 0.08 | 0.00 | 0.06 | 0.02 | 0.00 | 0.00 |  |  |  |  |  |  |  |  |  |
| PHQ8 | 0.05 | 0.00 | 0.00 | 0.05 | 0.05 | 0.13 | 0.15 |  |  |  |  |  |  |  |  |
| GAD1 | 0.04 | 0.13 | 0.00 | 0.11 | 0.00 | 0.04 | 0.00 | 0.00 |  |  |  |  |  |  |  |
| GAD2 | 0.00 | 0.04 | 0.04 | 0.00 | 0.00 | 0.08 | 0.00 | 0.00 | 0.33 |  |  |  |  |  |  |
| GAD3 | 0.01 | 0.06 | 0.00 | 0.00 | 0.03 | 0.15 | 0.05 | 0.10 | 0.11 | 0.16 |  |  |  |  |  |
| GAD4 | 0.00 | 0.03 | 0.02 | 0.00 | 0.00 | 0.01 | 0.06 | 0.03 | 0.09 | 0.10 | 0.12 |  |  |  |  |
| GAD5 | 0.00 | 0.00 | 0.00 | 0.05 | 0.06 | 0.03 | 0.04 | 0.06 | 0.00 | 0.13 | 0.00 | 0.18 |  |  |  |
| GAD6 | 0.00 | 0.08 | 0.00 | 0.06 | 0.15 | 0.05 | 0.05 | 0.04 | 0.02 | 0.17 | 0.02 | 0.21 | 0.14 |  |  |
| GAD7 | 0.01 | 0.00 | 0.02 | 0.01 | 0.12 | 0.13 | 0.00 | 0.00 | 0.00 | 0.00 | 0.13 | 0.00 | 0.02 | 0.13 |  |

*Note.* PHQ1 = Anhedonia, PHQ2 = Mood, PHQ3 = Sleep, PHQ4 = Energy, PHQ5 = Appetite, PHQ6 = Guilty, PHQ7 = Concentration, PHQ8 = Motor, GAD1 = Nervous, GAD2 = Control worry, GAD3 = Too much worry, GAD4 = Relax, GAD5 = Restless, GAD6 = Irritable, GAD7 = Afraid.

Table S5 The matrix of edge weights of the low adaptability group’s symptom network.

|  | PHQ1 | PHQ2 | PHQ3 | PHQ4 | PHQ5 | PHQ6 | PHQ7 | PHQ8 | GAD1 | GAD2 | GAD3 | GAD4 | GAD5 | GAD6 | GAD7 |
| --- | --- | --- | --- | --- | --- | --- | --- | --- | --- | --- | --- | --- | --- | --- | --- |
| PHQ1 |  |  |  |  |  |  |  |  |  |  |  |  |  |  |  |
| PHQ2 | 0.14 |  |  |  |  |  |  |  |  |  |  |  |  |  |  |
| PHQ3 | 0.00 | 0.13 |  |  |  |  |  |  |  |  |  |  |  |  |  |
| PHQ4 | 0.24 | 0.13 | 0.06 |  |  |  |  |  |  |  |  |  |  |  |  |
| PHQ5 | 0.00 | 0.00 | 0.19 | 0.07 |  |  |  |  |  |  |  |  |  |  |  |
| PHQ6 | 0.06 | 0.26 | 0.00 | 0.09 | 0.13 |  |  |  |  |  |  |  |  |  |  |
| PHQ7 | 0.12 | 0.00 | 0.06 | 0.00 | 0.13 | 0.02 |  |  |  |  |  |  |  |  |  |
| PHQ8 | 0.05 | 0.02 | 0.10 | 0.02 | 0.05 | 0.04 | 0.09 |  |  |  |  |  |  |  |  |
| GAD1 | 0.05 | 0.18 | 0.02 | 0.03 | 0.01 | 0.08 | 0.04 | -0.03 |  |  |  |  |  |  |  |
| GAD2 | 0.00 | 0.04 | 0.00 | 0.01 | 0.00 | 0.06 | 0.02 | 0.02 | 0.32 |  |  |  |  |  |  |
| GAD3 | 0.00 | 0.04 | 0.00 | 0.00 | 0.08 | 0.01 | 0.03 | 0.04 | 0.14 | 0.26 |  |  |  |  |  |
| GAD4 | 0.00 | 0.00 | 0.15 | 0.12 | 0.00 | 0.00 | 0.07 | 0.01 | 0.08 | 0.15 | 0.22 |  |  |  |  |
| GAD5 | 0.05 | 0.00 | 0.00 | -0.05 | 0.06 | 0.00 | 0.05 | 0.17 | 0.00 | 0.00 | 0.07 | 0.13 |  |  |  |
| GAD6 | 0.04 | 0.04 | 0.05 | 0.10 | 0.00 | 0.07 | 0.02 | 0.11 | 0.02 | 0.11 | 0.11 | 0.02 | 0.19 |  |  |
| GAD7 | -0.04 | 0.01 | 0.01 | 0.00 | 0.07 | 0.19 | 0.00 | 0.04 | 0.00 | 0.00 | 0.05 | 0.08 | 0.16 | 0.18 |  |

*Note.* PHQ1 = Anhedonia, PHQ2 = Mood, PHQ3 = Sleep, PHQ4 = Energy, PHQ5 = Appetite, PHQ6 = Guilty, PHQ7 = Concentration, PHQ8 = Motor, GAD1 = Nervous, GAD2 = Control worry, GAD3 = Too much worry, GAD4 = Relax, GAD5 = Restless, GAD6 = Irritable, GAD7 = Afraid.

Table S6 The comparison of edges between HGN and LGN.

| Edge | W | *p* | *p_c_* |
| --- | --- | --- | --- |
| PHQ6-GAD3 | 0.14 | 0.05 | 0.68 |
| PHQ3-GAD4 | 0.13 | 0.05 | 0.68 |
| PHQ4-GAD5 | 0.10 | 0.03 | 0.68 |
| GAD2-GAD5 | 0.13 | 0.01 | 0.49 |
| PHQ5-GAD6 | 0.15 | 0.01 | 0.49 |
| GAD4-GAD6 | 0.19 | 0.01 | 0.49 |

*Note.* HGN = The high adaptability group’s symptom network, LGN = The low adaptability group’s symptom network, W = The differences of edge weight between two networks, PHQ3 = Sleep, PHQ4 = Energy, PHQ5 = Appetite, PHQ6 = Guilty, GAD2 = Control worry, GAD3 = Too much worry, GAD4 = Relax, GAD5 = Restless, GAD6 = Irritable, *p* = The uncorrected p-value, *p_c_* = The p-value after the False Discovery Rate (FDR) correction.

Table S7 The comparison of nodes between HGN and LGN.

|  | EI | *p_c_* | bridgeEI | *p_c_* |
| --- | --- | --- | --- | --- |
| PHQ3 | -0.32 | 0.01 | -0.15 | 0.61 |

*Note.* HGN = The high adaptability group’s symptom network, LGN = The low adaptability group’s symptom network, EI = The difference in experience influence between two networks, bridge EI = The difference in bridge experience influence between the two networks, PHQ3 = Sleep, *p_c_* = The p-value after the False Discovery Rate (FDR) correction.

Table S8 The matrix of edge weights of the symptom network at T1.

|  | PHQ1 | PHQ2 | PHQ3 | PHQ4 | PHQ5 | PHQ6 | PHQ7 | PHQ8 | GAD1 | GAD2 | GAD3 | GAD4 | GAD5 | GAD6 | GAD7 |
| --- | --- | --- | --- | --- | --- | --- | --- | --- | --- | --- | --- | --- | --- | --- | --- |
| PHQ1 |  |  |  |  |  |  |  |  |  |  |  |  |  |  |  |
| PHQ2 | 0.15 |  |  |  |  |  |  |  |  |  |  |  |  |  |  |
| PHQ3 | 0.09 | 0.12 |  |  |  |  |  |  |  |  |  |  |  |  |  |
| PHQ4 | 0.19 | 0.11 | 0.00 |  |  |  |  |  |  |  |  |  |  |  |  |
| PHQ5 | 0.00 | 0.00 | 0.17 | 0.14 |  |  |  |  |  |  |  |  |  |  |  |
| PHQ6 | 0.09 | 0.27 | 0.02 | 0.09 | 0.11 |  |  |  |  |  |  |  |  |  |  |
| PHQ7 | 0.13 | 0.00 | 0.00 | 0.00 | 0.09 | 0.07 |  |  |  |  |  |  |  |  |  |
| PHQ8 | 0.12 | 0.00 | 0.05 | 0.01 | 0.03 | 0.05 | 0.15 |  |  |  |  |  |  |  |  |
| GAD1 | 0.10 | 0.15 | 0.04 | 0.06 | -0.06 | 0.07 | 0.00 | -0.10 |  |  |  |  |  |  |  |
| GAD2 | 0.00 | 0.02 | 0.03 | 0.00 | 0.00 | 0.07 | 0.00 | 0.00 | 0.32 |  |  |  |  |  |  |
| GAD3 | 0.02 | 0.06 | 0.00 | 0.02 | 0.06 | 0.02 | 0.09 | 0.07 | 0.09 | 0.20 |  |  |  |  |  |
| GAD4 | -0.05 | 0.01 | 0.07 | 0.08 | 0.01 | 0.02 | 0.07 | 0.05 | 0.11 | 0.15 | 0.17 |  |  |  |  |
| GAD5 | 0.01 | 0.00 | -0.03 | 0.00 | 0.06 | 0.01 | 0.02 | 0.20 | 0.00 | 0.05 | 0.05 | 0.15 |  |  |  |
| GAD6 | 0.00 | 0.05 | 0.09 | 0.14 | 0.06 | 0.06 | 0.03 | 0.14 | 0.09 | 0.16 | 0.05 | 0.06 | 0.14 |  |  |
| GAD7 | 0.00 | 0.02 | 0.09 | 0.00 | 0.08 | 0.15 | -0.01 | 0.00 | -0.04 | 0.00 | 0.07 | 0.10 | 0.14 | 0.16 |  |

*Note.* PHQ1 = Anhedonia, PHQ2 = Mood, PHQ3 = Sleep, PHQ4 = Energy, PHQ5 = Appetite, PHQ6 = Guilty, PHQ7 = Concentration, PHQ8 = Motor, GAD1 = Nervous, GAD2 = Control worry, GAD3 = Too much worry, GAD4 = Relax, GAD5 = Restless, GAD6 = Irritable, GAD7 = Afraid.

Table S9 The matrix of edge weights of the symptom network at T2.

|  | PHQ1 | PHQ2 | PHQ3 | PHQ4 | PHQ5 | PHQ6 | PHQ7 | PHQ8 | GAD1 | GAD2 | GAD3 | GAD4 | GAD5 | GAD6 | GAD7 |
| --- | --- | --- | --- | --- | --- | --- | --- | --- | --- | --- | --- | --- | --- | --- | --- |
| PHQ1 |  |  |  |  |  |  |  |  |  |  |  |  |  |  |  |
| PHQ2 | 0.24 |  |  |  |  |  |  |  |  |  |  |  |  |  |  |
| PHQ3 | 0.09 | 0.21 |  |  |  |  |  |  |  |  |  |  |  |  |  |
| PHQ4 | 0.24 | 0.18 | 0.10 |  |  |  |  |  |  |  |  |  |  |  |  |
| PHQ5 | 0.01 | 0.00 | 0.14 | 0.18 |  |  |  |  |  |  |  |  |  |  |  |
| PHQ6 | 0.00 | 0.24 | 0.01 | 0.07 | 0.16 |  |  |  |  |  |  |  |  |  |  |
| PHQ7 | 0.07 | 0.00 | 0.00 | 0.00 | 0.08 | 0.13 |  |  |  |  |  |  |  |  |  |
| PHQ8 | 0.11 | 0.00 | 0.00 | 0.01 | 0.12 | 0.08 | 0.12 |  |  |  |  |  |  |  |  |
| GAD1 | 0.00 | 0.12 | 0.05 | 0.11 | 0.00 | 0.03 | 0.00 | 0.04 |  |  |  |  |  |  |  |
| GAD2 | 0.00 | 0.03 | 0.00 | 0.03 | 0.03 | 0.00 | 0.01 | 0.00 | 0.21 |  |  |  |  |  |  |
| GAD3 | 0.03 | 0.06 | 0.00 | 0.00 | 0.00 | 0.07 | 0.01 | 0.00 | 0.16 | 0.30 |  |  |  |  |  |
| GAD4 | 0.02 | 0.02 | 0.00 | 0.01 | 0.05 | 0.03 | 0.00 | 0.06 | 0.12 | 0.21 | 0.21 |  |  |  |  |
| GAD5 | 0.00 | 0.00 | 0.00 | 0.00 | 0.05 | 0.00 | 0.09 | 0.18 | 0.00 | 0.01 | 0.01 | 0.26 |  |  |  |
| GAD6 | 0.04 | 0.00 | 0.01 | 0.01 | 0.00 | 0.12 | 0.06 | 0.08 | 0.11 | 0.07 | 0.12 | 0.08 | 0.15 |  |  |
| GAD7 | 0.05 | 0.05 | 0.05 | 0.00 | 0.00 | 0.05 | 0.06 | 0.02 | 0.00 | 0.08 | 0.10 | 0.02 | 0.15 | 0.15 |  |

*Note.* PHQ1 = Anhedonia, PHQ2 = Mood, PHQ3 = Sleep, PHQ4 = Energy, PHQ5 = Appetite, PHQ6 = Guilty, PHQ7 = Concentration, PHQ8 = Motor, GAD1 = Nervous, GAD2 = Control worry, GAD3 = Too much worry, GAD4 = Relax, GAD5 = Restless, GAD6 = Irritable, GAD7 = Afraid.

Table S10 The predictability for each node in the two networks.

|  | T1  (N = 426) | T2  (N = 426) |
| --- | --- | --- |
| PHQ1 | 0.3896 | 0.5409 |
| PHQ2 | 0.4933 | 0.6818 |
| PHQ3 | 0.3851 | 0.4272 |
| PHQ4 | 0.4277 | 0.5771 |
| PHQ5 | 0.3272 | 0.4707 |
| PHQ6 | 0.5296 | 0.5708 |
| PHQ7 | 0.2605 | 0.3510 |
| PHQ8 | 0.4061 | 0.4991 |
| GAD1 | 0.5489 | 0.6537 |
| GAD2 | 0.5756 | 0.7084 |
| GAD3 | 0.4747 | 0.7325 |
| GAD4 | 0.5283 | 0.7125 |
| GAD5 | 0.4056 | 0.5826 |
| GAD6 | 0.6013 | 0.5985 |
| GAD7 | 0.3916 | 0.5057 |

*Note.* T1 = The symptom network at T1, T2 = The symptom network at T2, PHQ1 = Anhedonia, PHQ2 = Mood, PHQ3 = Sleep, PHQ4 = Energy, PHQ5 = Appetite, PHQ6 = Guilty, PHQ7 = Concentration, PHQ8 = Motor, GAD1 = Nervous, GAD2 = Control worry, GAD3 = Too much worry, GAD4 = Relax, GAD5 = Restless, GAD6 = Irritable, GAD7 = Afraid.

Table S11 The comparison of edges between symptom networks at T2 and T1.

| Edge | W | *p* | *p_c_* |
| --- | --- | --- | --- |
| PHQ1-GAD1 | 0.10 | 4.00 × 10^-3^ | 0.21 |
| PHQ8-GAD1 | 0.14 | 9.99 × 10^-4^ | 0.10 |
| PHQ8-GAD3 | 0.07 | 0.03 | 0.71 |
| PHQ1-GAD4 | 0.07 | 0.02 | 0.71 |

*Note.* W = The differences of edge weight between two networks, PHQ1 = Anhedonia, PHQ8 = Motor, GAD1 = Nervous, GAD3 = Too much worry, GAD4 = Relax, *p* = The uncorrected p-value, *p_c_* = The p-value after the False Discovery Rate (FDR) correction.

Table S12 The comparison of nodes between symptom networks at T2 and T1.

| Node | EI | *p* | bridgeEI | *p* |
| --- | --- | --- | --- | --- |
| PHQ2 | 0.19 | 0.02 | -0.03 | 0.76 |
| PHQ3 | -0.10 | 0.20 | -0.20 | 0.03 |
| GAD6 | -0.23 | 0.01 | -0.25 | 0.03 |

*Note.* EI = The differences in experience influence between two networks, bridge EI = The differences in bridge experience influence between the two networks (T2 – T1), PHQ2 = Mood, PHQ3 = Sleep, GAD6 = Irritable, *p* = The uncorrected p-value.

***
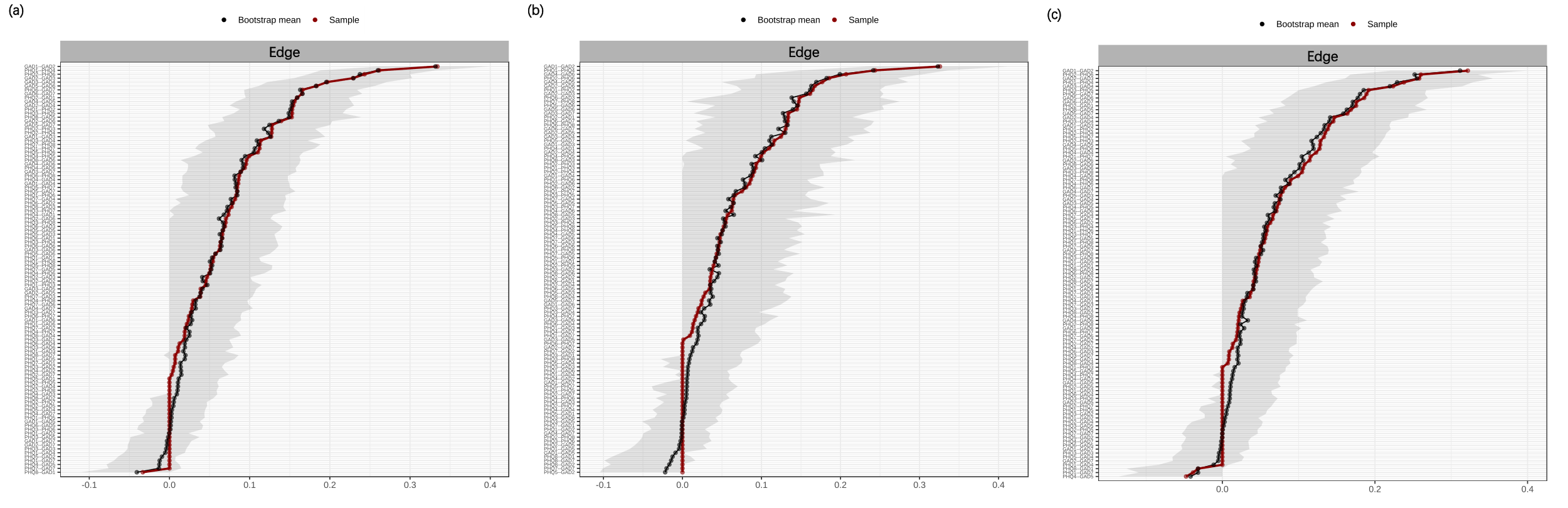
***

**Figure S1** The results for bootstrapped confidence intervals of estimated edge-weights. (a) The whole symptom network, (b) the high adaptability group’s symptom network, and (c) the low adaptability group’s symptom network.

***
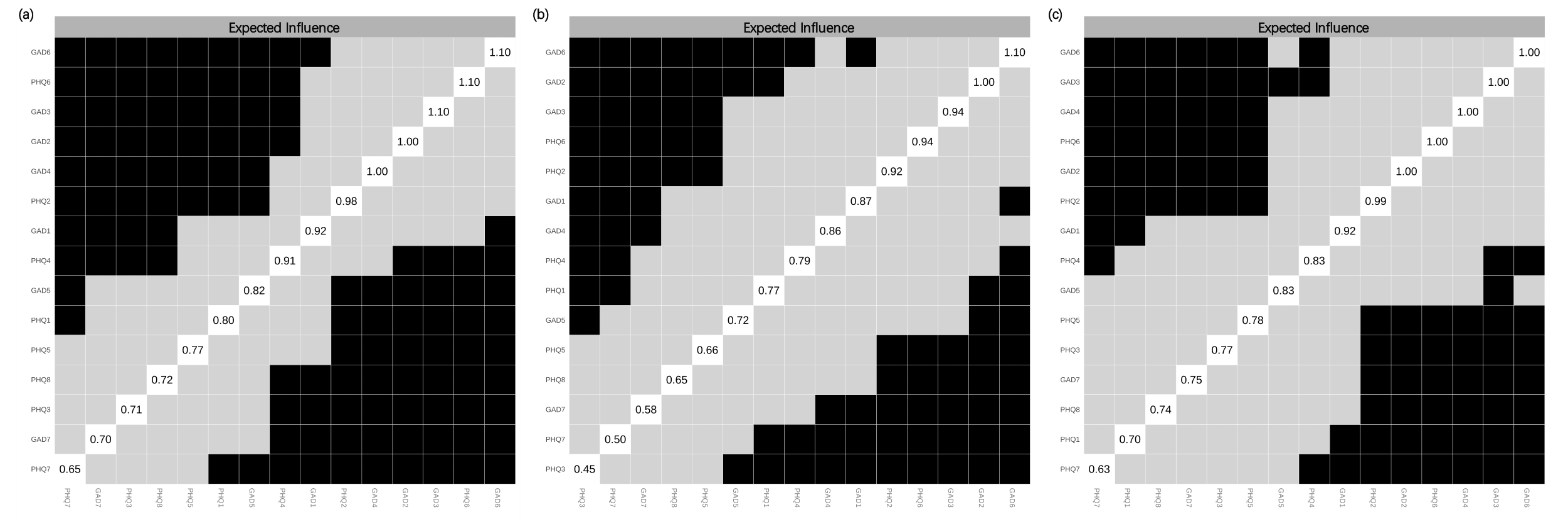
***

**Figure S2** The difference test results for expected influence. (a) The whole symptom network, (b) the high adaptability group’s symptom network, and (c) the low adaptability group’s symptom network. The black grid indicates a significant difference between the two corresponding nodes.


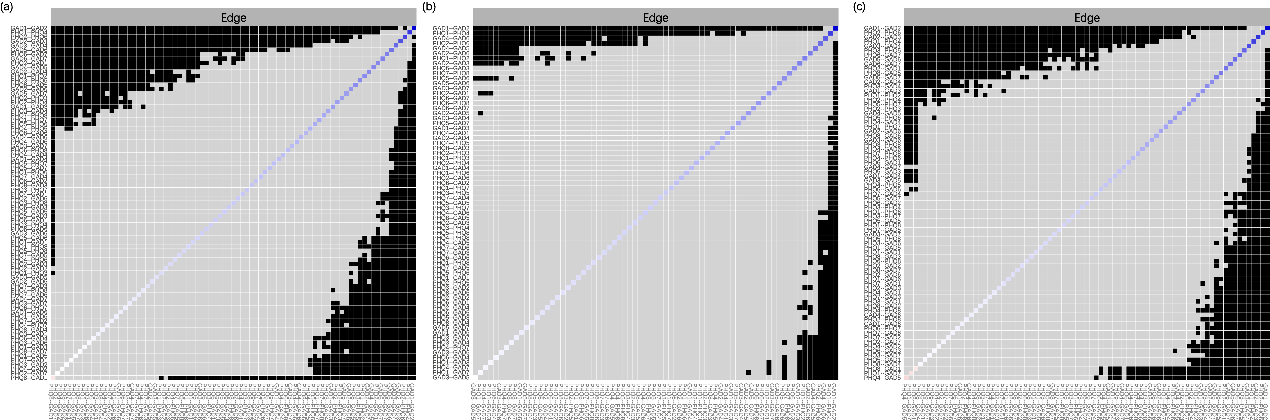


**Figure S3** The difference test results for edge weights. (a) The whole symptom network, (b) the high adaptability group’s symptom network, and (c) the low adaptability group’s symptom network. The black grid indicates a significant difference between the two corresponding edges.


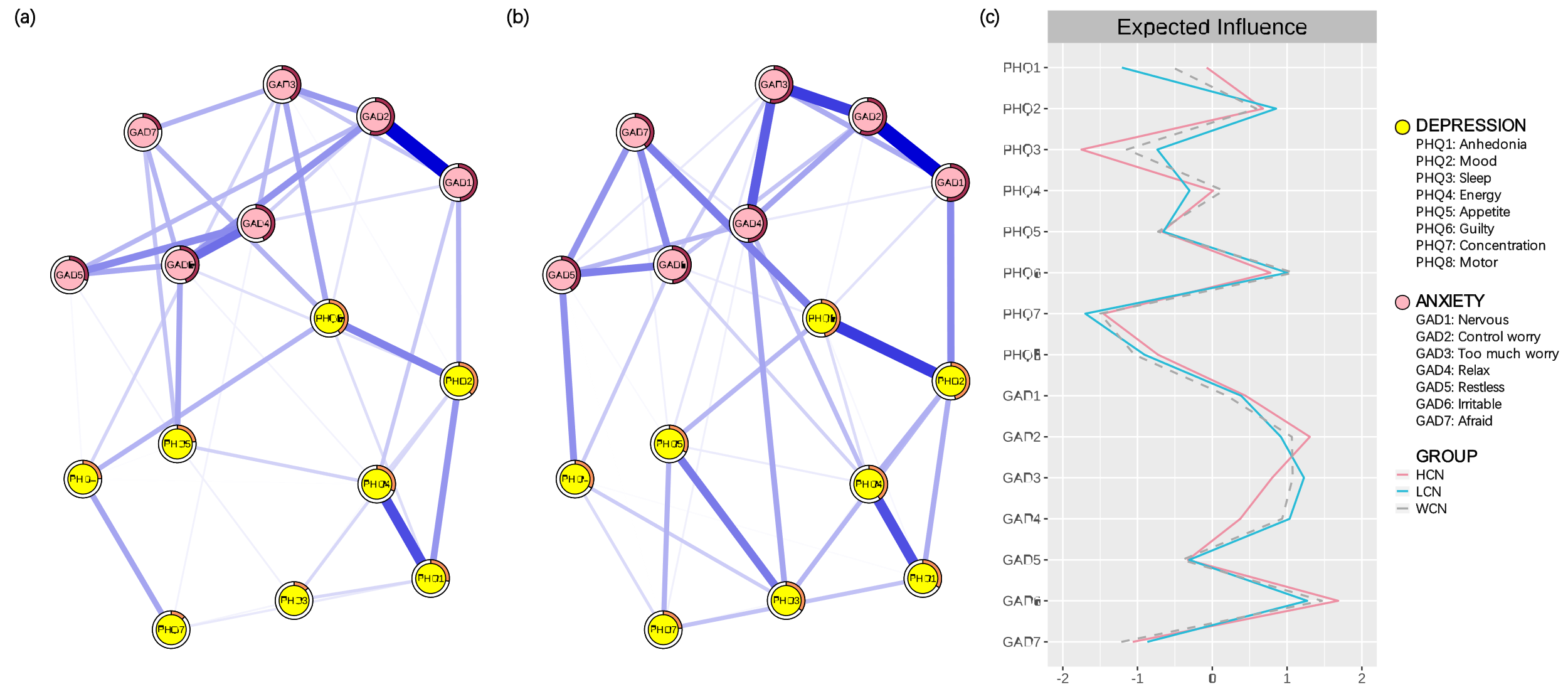


**Figure S4** The symptom networks for high and low adaptability groups (HGN and LGN) at T1 (N_HCN_ = 402, N_LCN_ = 431). Yellow nodes represent the symptoms of depression; pink nodes represent the symptoms of anxiety disorder. A wider edge signifies a greater weight between nodes. The ring-shaped pie charts represent the predictability. (a) The structure of HGN. The first three strongest symptoms are Irritable (EI = 1.10), Control worry (EI = 1.04), and Too much worry (EI = 0.94). (b) The structure of LGN. The first three strongest symptoms are Irritable (EI = 1.05), Too much worry (EI = 1.04), and Relax (EI = 1.02). (c) The contrast of expected influence (EI) between three networks. This index reflects the importance of a node in the network. The values are Z‐standardized.


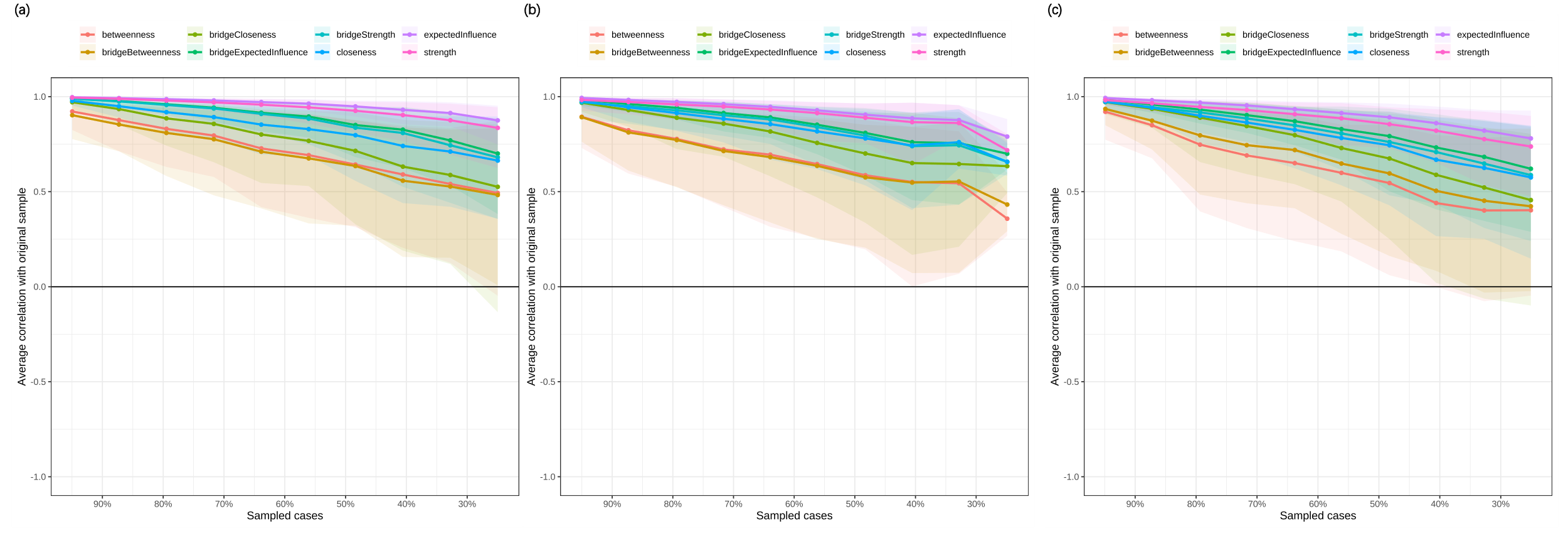


**Figure S5** The case-dropping results for all centrality indices. (a) The whole symptom network, (b) the high adaptability group’s symptom network, and (c) the low adaptability group’s symptom network.


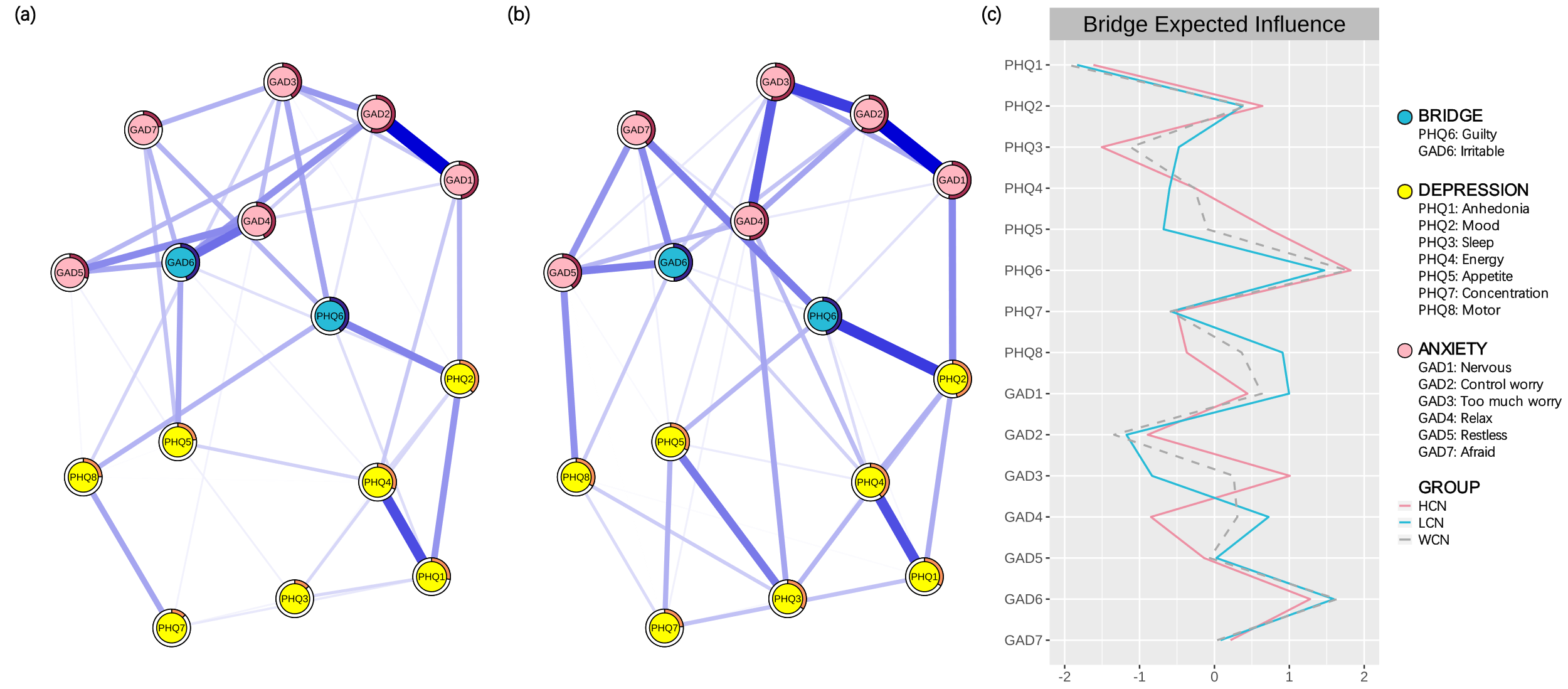


**Figure S6** The symptom networks for high and low adaptability groups (HGN and LGN) at T1 showing bridge symptoms (N_HCN_ = 402, N_LCN_ = 431). (a-b) The structures of HGN and LGN. Yellow nodes represent the symptoms of depression; pink nodes represent the symptoms of anxiety disorder; blue nodes represent the bridge symptoms. Guilty and Irritable are the bridge symptoms in both networks. (c) The contrast of bridge expected influence between three networks. This index reflects the extent to which a node acts as a bridge symptom. The values are Z‐standardized.


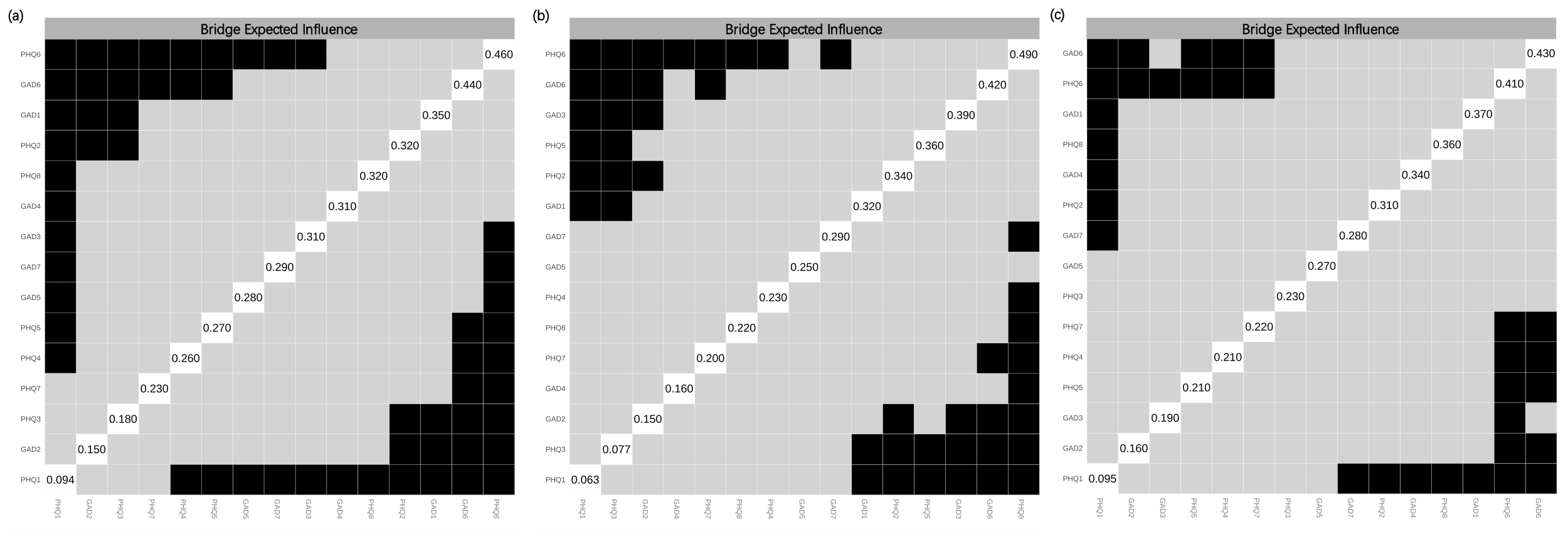


**Figure S7** The difference test results for bridge EI. (a) The whole symptom network, (b) the high adaptability group’s symptom network and, (c) the low adaptability group’s symptom network. The black grid indicates a significant difference between the two corresponding nodes.

***
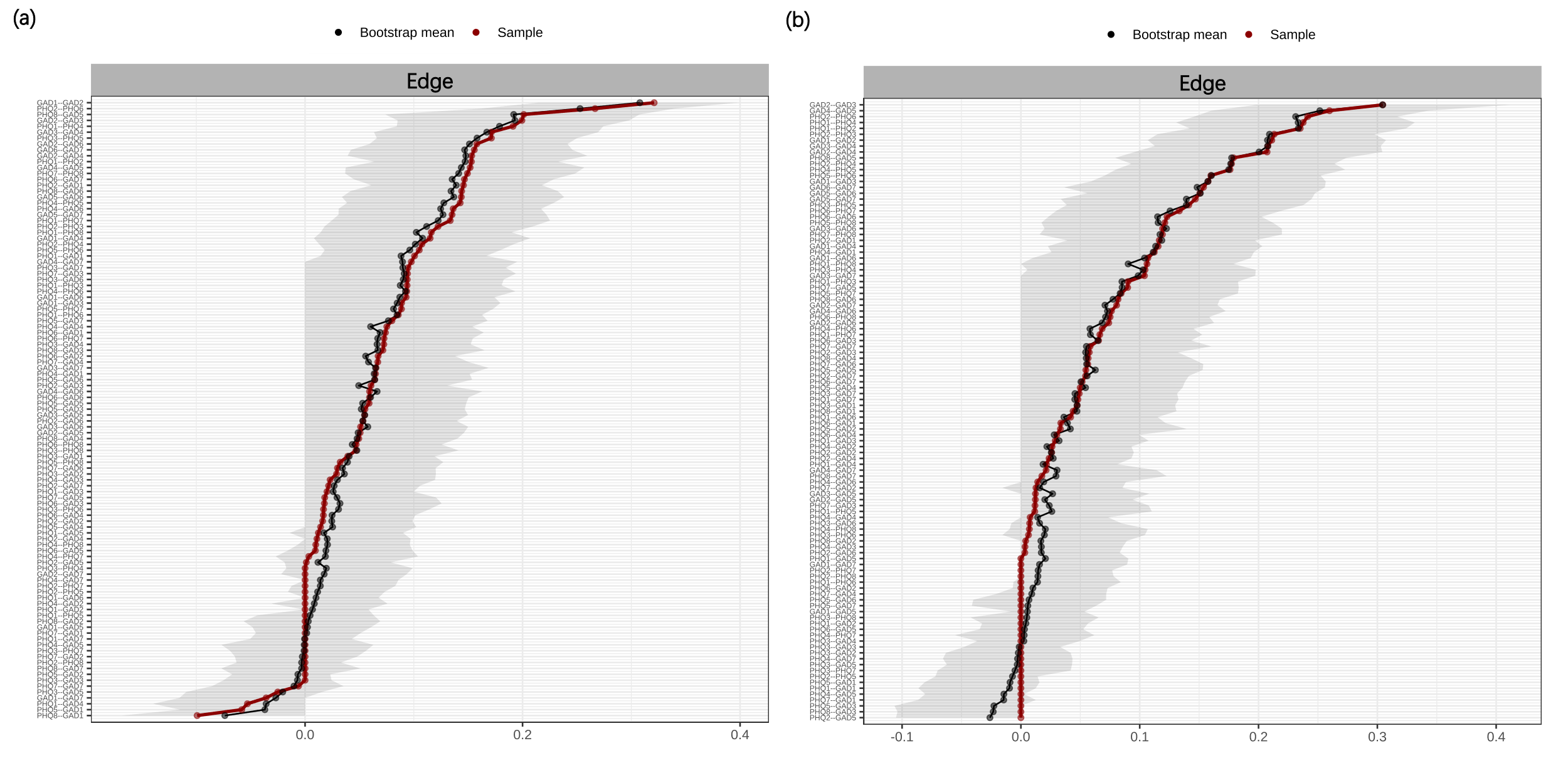
***

**Figure S8** The results for bootstrapped confidence intervals of estimated edge-weights. (a) The symptom network at T1 and (b) the symptom network at T2.

***
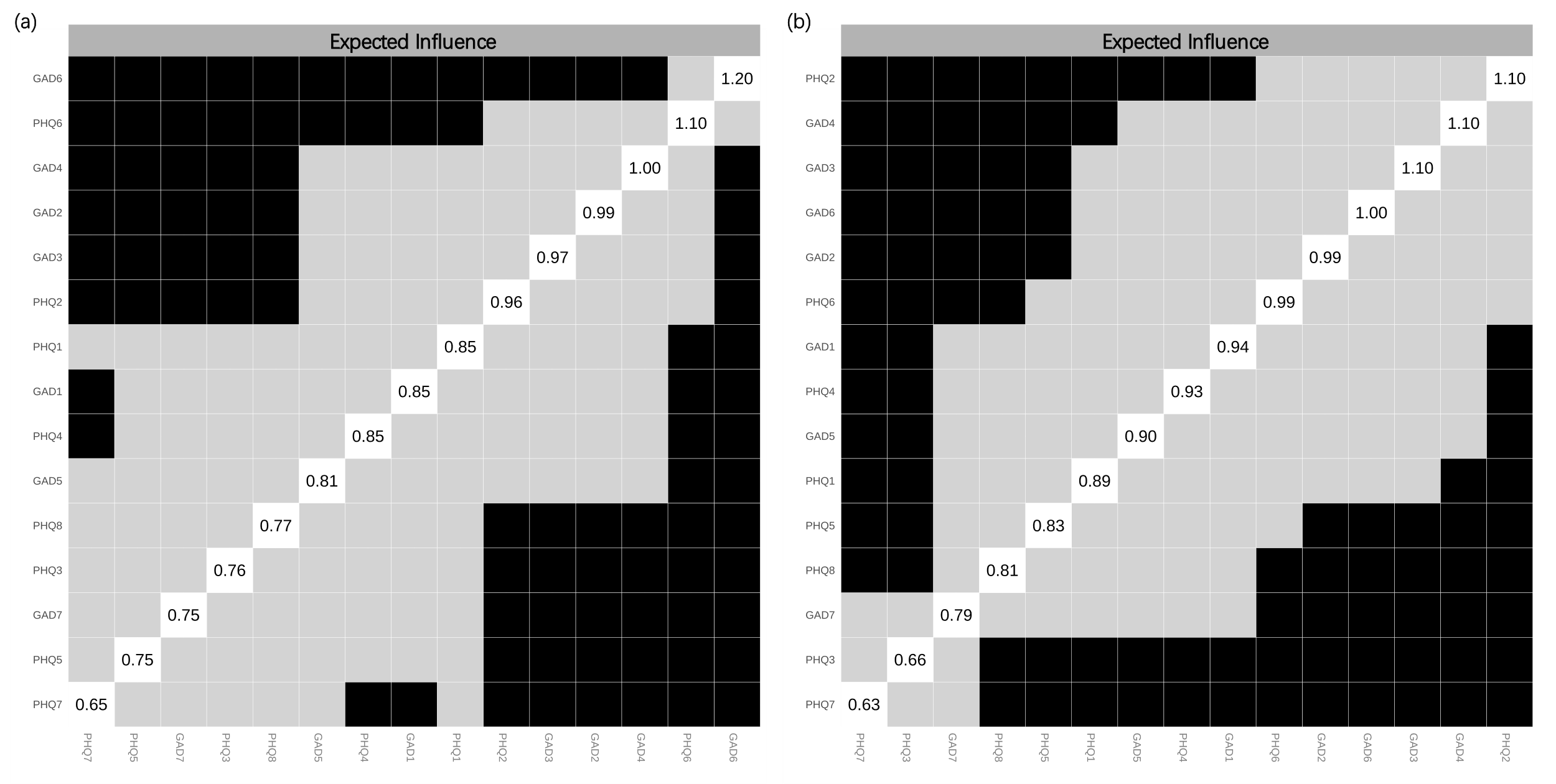
***

**Figure S9** The difference test results for expected influence. (a) The symptom network at T1 and (b) the symptom network at T2. The black grid indicates a significant difference between the two corresponding nodes.


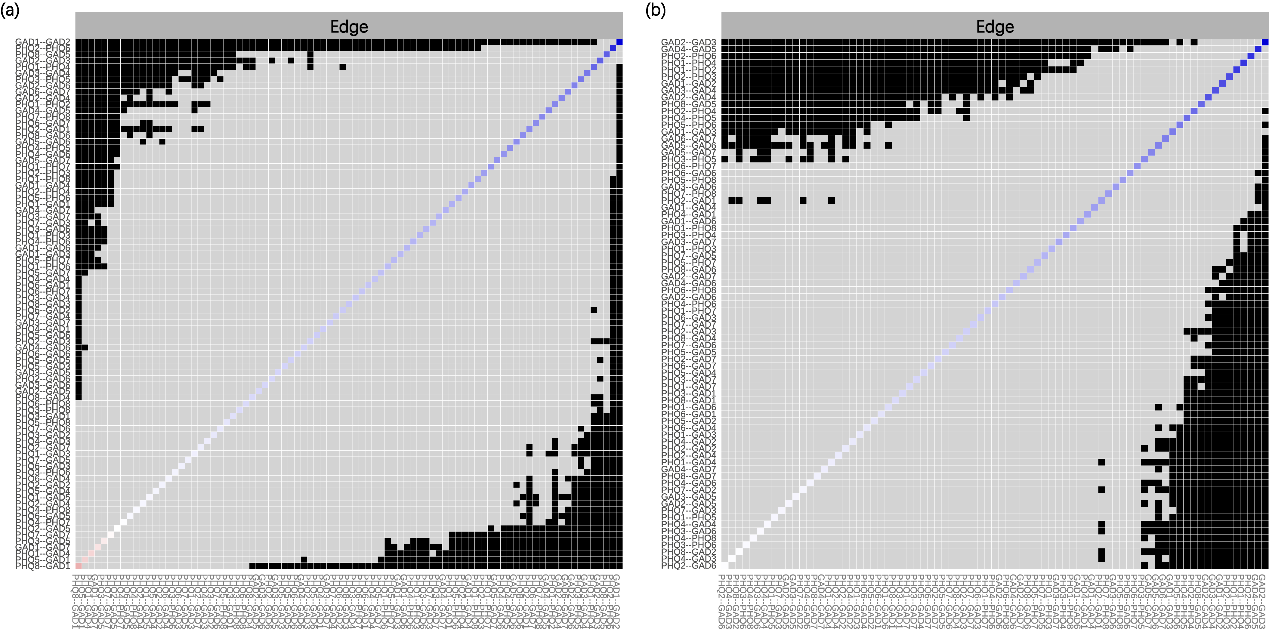


**Figure S10** The difference test results for edge weights. (a) The symptom network at T1 and (b) the symptom network at T2. The black grid indicates a significant difference between the two corresponding edges.

***
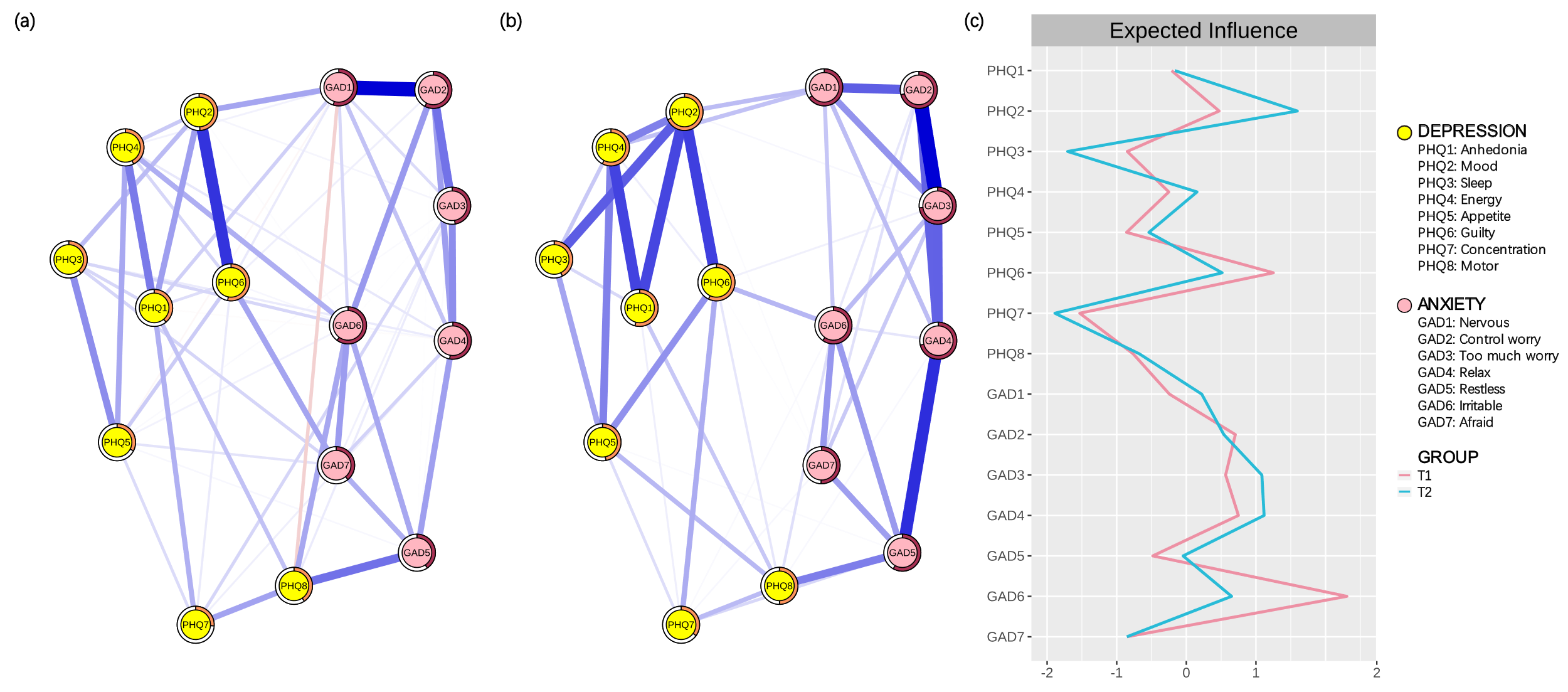
***

**Figure S11** The symptom networks at both timepoints (T1 and T2, N = 426). Yellow nodes represent the symptoms of depression; pink nodes represent the symptoms of anxiety disorder. A wider edge signifies a greater weight between nodes. The ring-shaped pie charts represent the predictability. (a) The network at T1. The first three strongest symptoms are Irritable (EI = 1.24), Guilty (EI = 1.08), and Relax (EI = 1.00). (b) The network at T2. The first three strongest symptoms are Mood (EI = 1.15), Relax (EI = 1.08), and Too much worry (EI = 1.07). (c) The contrast of expected influence (EI) between two networks. This index reflects the importance of a node in the network. The values are Z‐standardized.

***
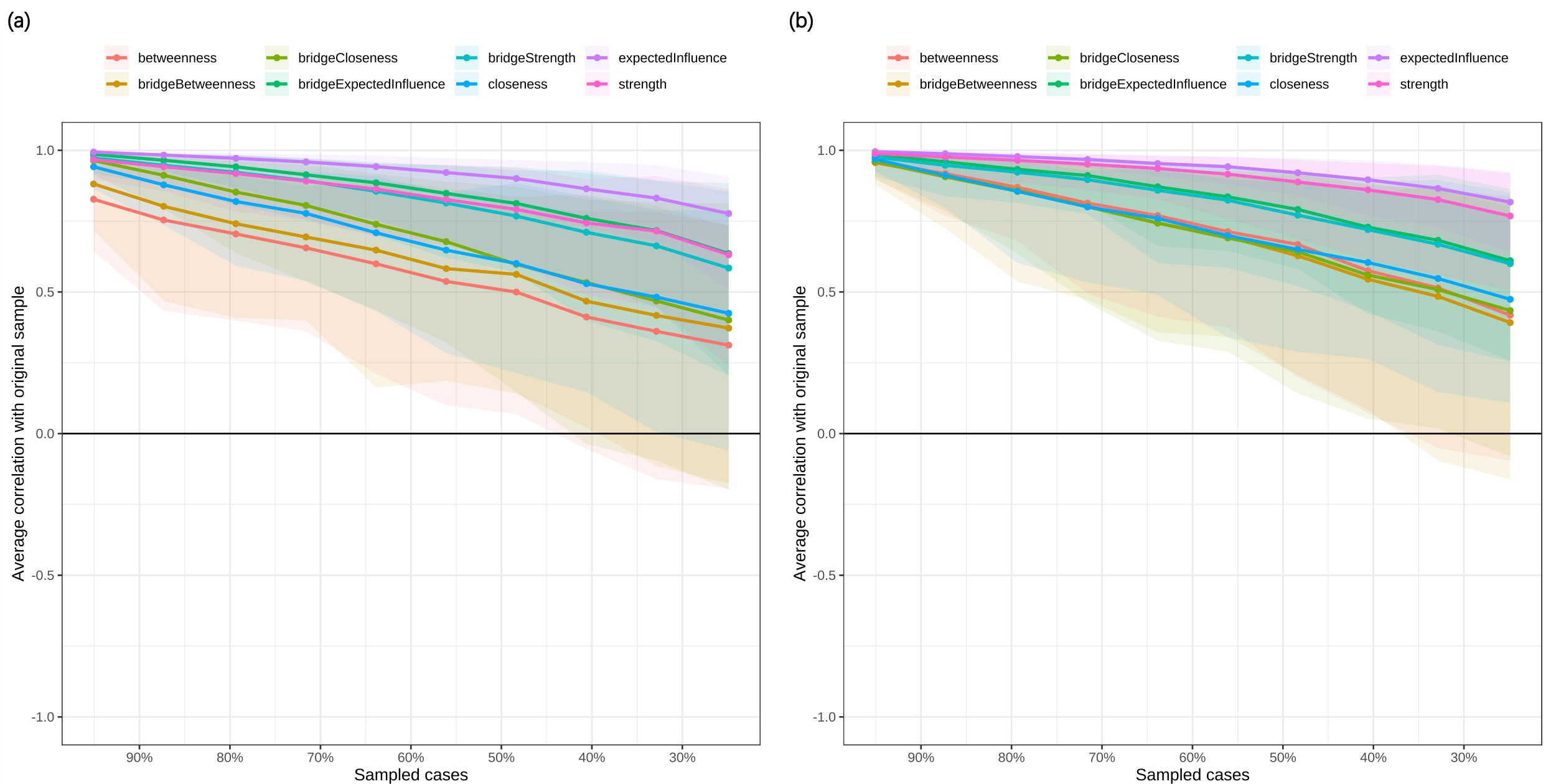
***

**Figure S12** The case-dropping results for all centrality indices. (a) The symptom network at T1 and (b) the symptom network at T2.


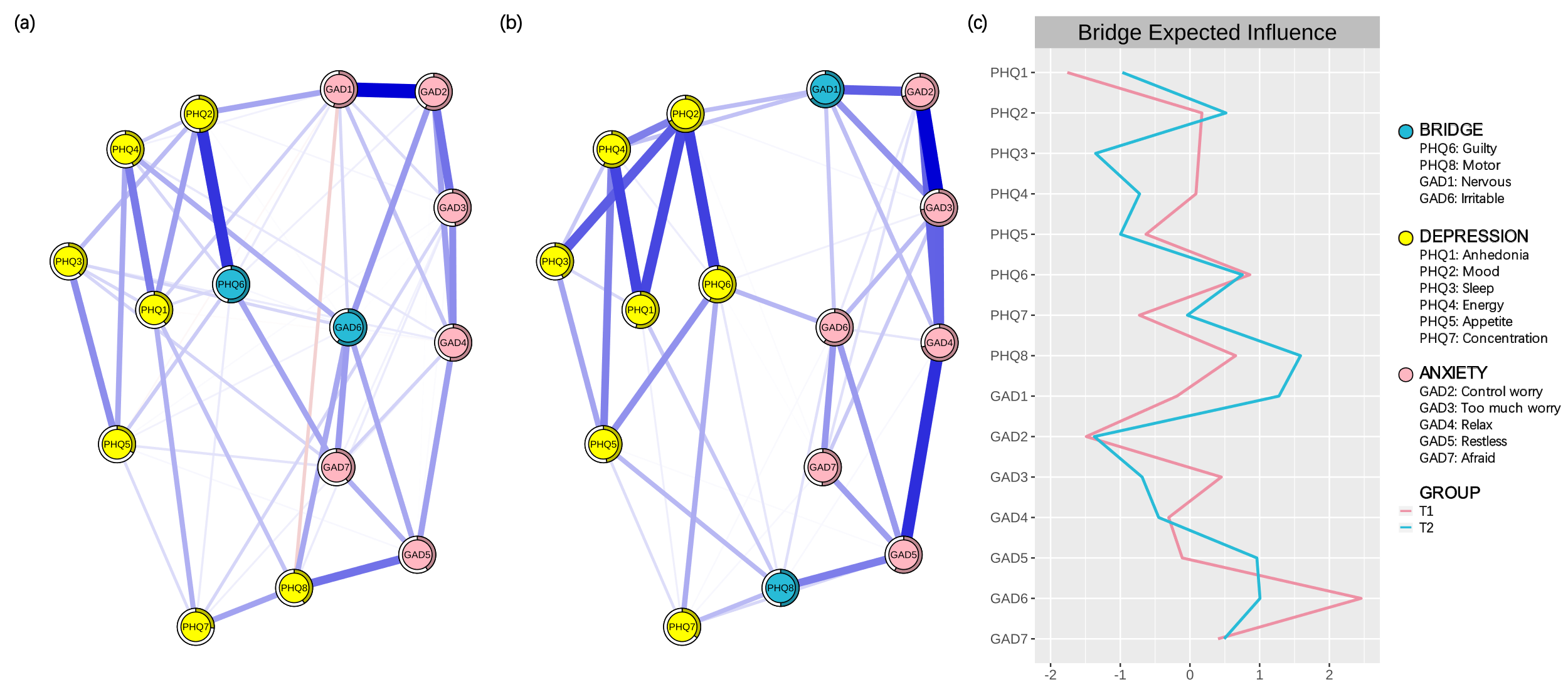


**Figure S13** The symptom networks at both timepoints (T1 and T2, N = 426) showing bridge symptoms. Yellow nodes represent the symptoms of depression; pink nodes represent the symptoms of anxiety disorder; blue nodes represent the bridge symptoms. (a) The network at T1. Guilty and Irritable are the bridge symptoms. (b) The network at T2. Motor and Nervous are the bridge symptoms. (c) The contrast of bridge expected influence between two networks. This index reflects the extent to which a node acts as a bridge symptom. The values are Z‐standardized.

***
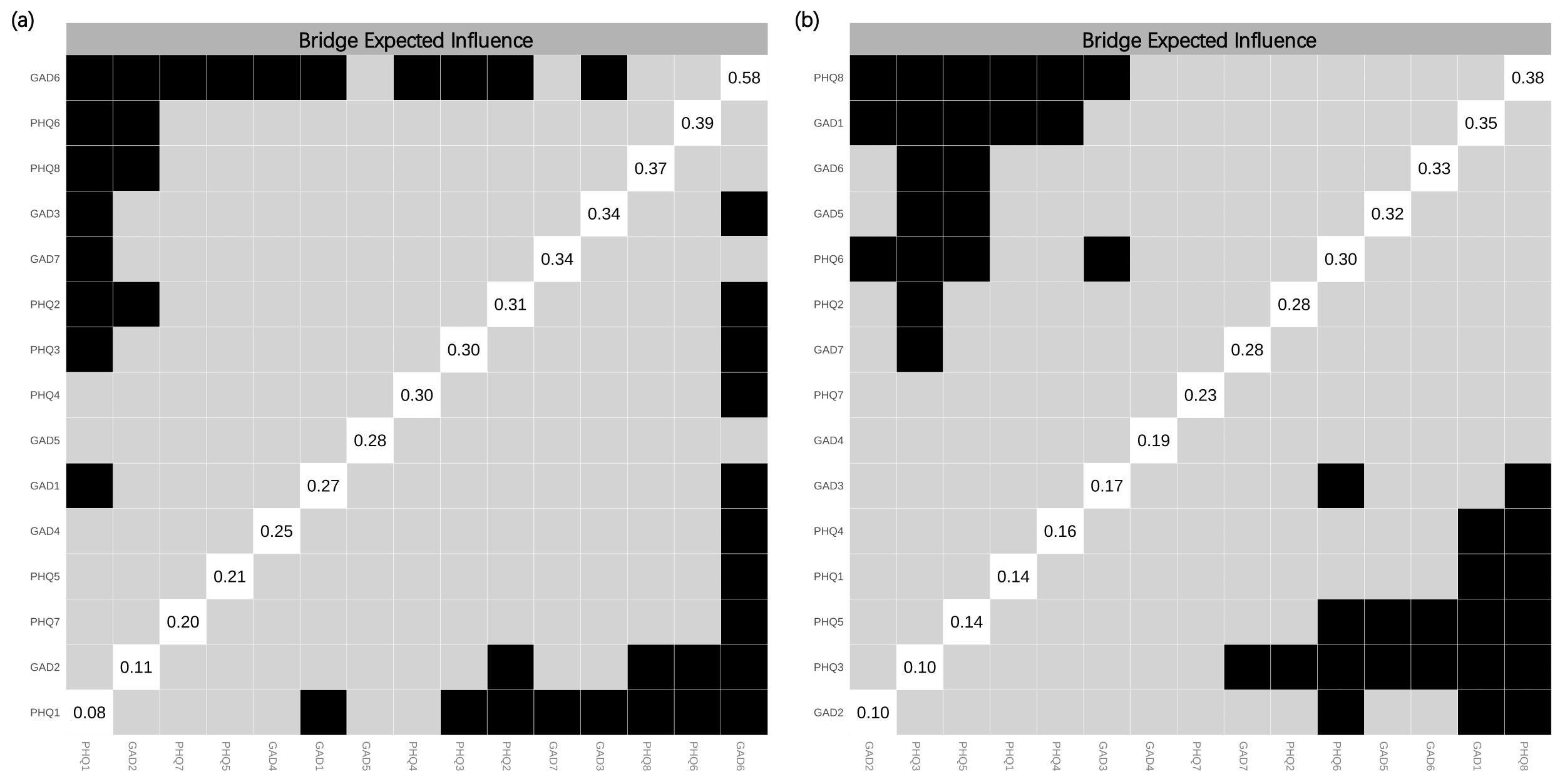
***

**Figure S14** The difference test results for bridge EI. (a) The symptom network at T1 and (b) the symptom network at T2. The black grid indicates a significant difference between the two corresponding nodes.
